# Artère carotide interne aberrante: une rare cause de toux (à propos d’un cas clinique)

**DOI:** 10.11604/pamj.2021.39.219.30266

**Published:** 2021-07-29

**Authors:** Asmaou Bouba Dalil, Antoine Bola Siafa, Yves Christian Andjock Nkouo, Roger Christian Meva’a Biouele, Esthelle Geneviève Minka Ngom, Jean Valentin Fokouo Fogha, Charles Mve Mvondo, Louis Richard Njock

**Affiliations:** 1 Hôpital Général de Yaoundé, Yaoundé, Cameroun,; 2 Centre Hospitalier Universitaire de Yaoundé, Yaoundé, Cameroun,; 3 Hôpital Central de Yaoundé, Yaoundé, Cameroun,; 4 Hôpital Général de Douala, Douala, Cameroun,; 5 Hôpital Régional de Bertoua, Bertoua, Cameroun,; 6 Faculté de Médecine et des Sciences Pharmaceutiques de Douala, Douala, Cameroun

**Keywords:** carotide, aberrante, toux, cas clinique, à propos d’un cas, carotid, aberrant, cough, case report

## Abstract

Le trajet aberrant de l´artère carotide interne représente une malformation congénitale rare. Dans sa localisation cervicale, l´incidence est rapportée à 5%. Plusieurs cas d'hémorragie mortelle par lésion d´une artère carotide interne aberrante ont été signalés lors d'interventions chirurgicales pharyngées, liées à son ignorance en per opératoire. Cette affection doit donc être connue du praticien ORL. Nous rapportons un cas de carotide interne aberrante, manifestée par une toux chronique liée au contact de l´épiglotte avec la masse pharyngée réalisée par l´artère aberrante. La patiente a été prise en charge comme pneumopathie chronique sans succès. Cette condition anatomique particulière doit être envisagée devant une masse pharyngée avant tout geste invasif.

## Abstract (en)

Aberrant course of the internal carotid artery is a rare congenital malformation. Aberrant course of cervical internal carotid artery accounts for 5% of cases. Several cases of fatal hemorrhage due to an injury to the aberrant internal carotid artery have been observed in patients undergoing pharyngeal surgery procedures. This was associated with a lack of detection during surgery. Ear nose and throat (ENT) practitioner need to know it. We here report a case of aberrant internal carotid manifesting as chronic cough due to the contact of the epiglottis with the pharyngeal mass caused by the aberrant artery. The patient was treated for chronic lung disease without success. This particular anatomical condition should be suspected in patients with pharyngeal mass before any invasive procedure.

## Introduction

Le trajet aberrant de l´artère carotide interne (ACI) représente une malformation congénitale rare [1]. Dans sa localisation cervicale, l´incidence est rapportée à 5% [2]. Au début du XXe siècle, plusieurs cas d'hémorragie mortelle ont été signalés lors d'interventions chirurgicales pharyngées, liées à son ignorance [3]. Cette affection doit donc être connue du praticien oto-rhino-laryngologiste (ORL). Souvent asymptomatique, une artère carotide interne aberrante peut provoquer une gêne pharyngée. Nous rapportons un cas de trajet aberrant de l´artère carotide interne, révélé par une toux persistante.

## Patient et observation

**Informations sur le patient:** il s´agissait d´une patiente de 70 ans, sans antécédents contributifs, venue consulter pour toux sèche évoluant depuis 10 mois, souvent en quintes pouvant durer quelques minutes. De nombreuses consultations médicales avaient été réalisées, notamment chez plusieurs médecins généralistes puis pneumologues sans véritable succès devant une radiographie pulmonaire normale. La recherche de bacilles acido-alcoolo-résistants dans les crachats était elle aussi négative, éliminant une tuberculose pulmonaire. Une consultation ORL a été demandée à la suite de ce bilan.

**Figure 1:**
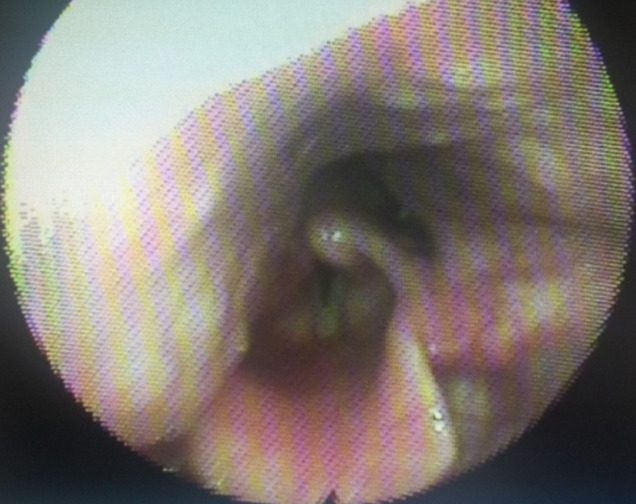
artère carotide interne aberrante réalisant une voussure pharyngée en contact avec le bord latéral droit de l´épiglotte

**Figure 2:**
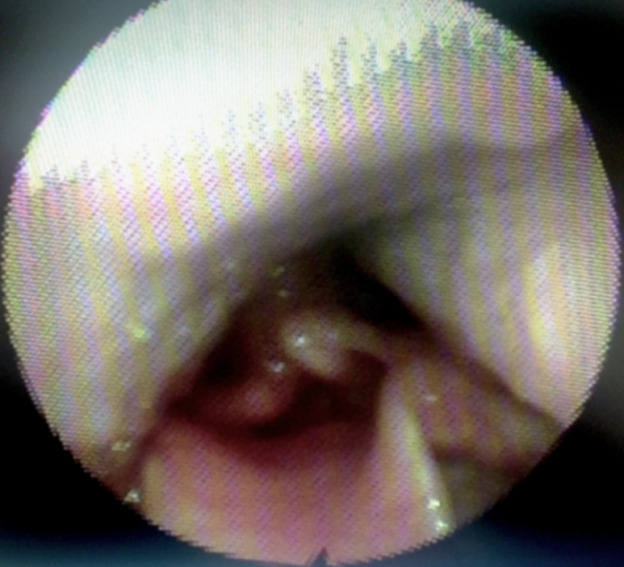
voussure controlatérale (gauche) sans contact avec l´épiglotte, luxation aryténoïdienne droite

**Trouvailles cliniques:** les examens oropharyngé, otoscopique et rhinoscopique étaient normaux. La nasofibroscopie a mis en évidence une voussure intéressant les parois pharyngées latérale droite et postérieure, en contact avec le bord latéral droit de l´épiglotte, et recouvrant quasi totalement l´aryténoïde et la zone des 3 replis droits (Figure 1). La toux était provoquée à chaque contact de la masse avec le bord latéral droit de l´épiglotte. Lors de la toux, on pouvait visualiser une luxation aryténoïdienne droite avec un aryténoïde inflammatoire. On observait également une voussure controlatérale de plus petite taille, cette dernière sans contact avec le larynx ou la zone des 3 replis (Figure 2).

**Figure 3:**
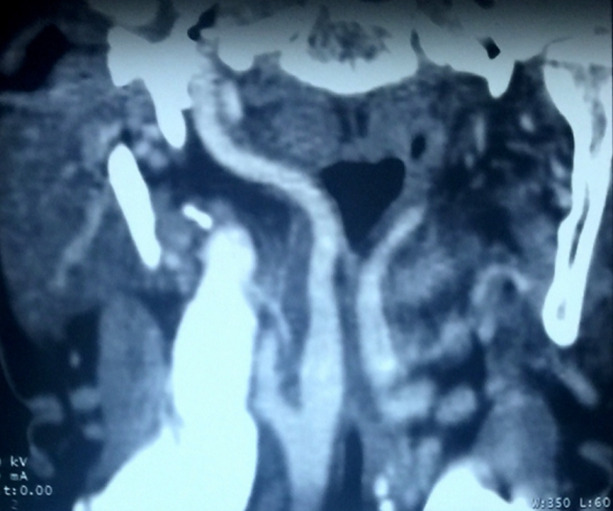
angio-TDM en coupe frontale: artère carotide interne droite et gauche aberrantes faisant protrusion dans le pharynx

**Figure 4:**
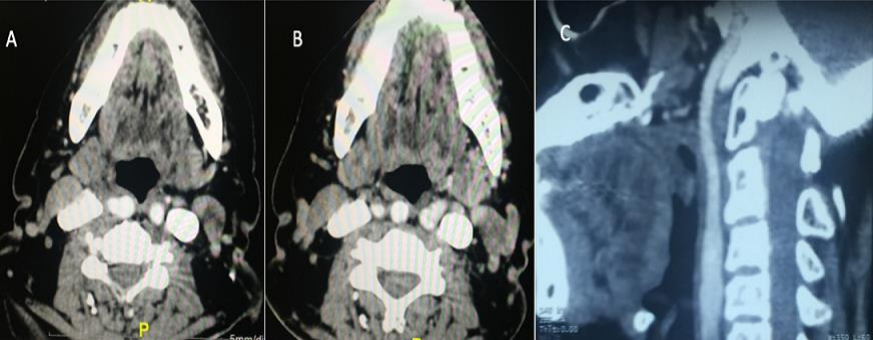
angio-TDM en coupes axiales et reconstruction sagittale: artère carotide interne tortueuse à droite (A) et à gauche (B), longeant la paroi pharyngée postérieure (C)

**Diagnostic:** une tomodensitométrie cervicale injectée, puis un angioscanner cervical ont permis de mettre en évidence une ACI aberrante et tortueuse faisant protrusion dans l´oropharynx et le pharyngolarynx de manière bilatérale, plus marquée à droite (Figure 3). Les ACI gauche et droite longeaient ensuite le mur pharyngé postérieur avant de rejoindre la base du crâne (Figure 4).

**Traitement:** l´abstention chirurgicale était de mise après concertation avec les chirurgiens vasculaires, des mesures posturales (patiente couchée sur le dos) et une surveillance annuelle avec nasofibroscopie de contrôle ont été préconisées.

**Evolution:** six mois plus tard, la toux restait présente, mais moins invalidante la nuit.

**Avis de la patiente:** la patiente était satisfaite de pouvoir mieux dormir la nuit en respectant les mesures posturales, malgré la persistance de la toux en journée.

**Consentement éclairé:** la patiente a donné son consentement écrit pour la publication de ses photos et informations médicales, son identité demeurant protégée. Elle a été informée de la pertinence de son cas et l´intérêt d´en faire une publication scientifique.

## Discussion

Les causes d'un trajet aberrant de l'artère carotide interne seraient en grande partie liées à une anomalie congénitale. L'artère carotide interne est dérivée de l´artère de la 3^e^ fente branchiale et de l´extrémité crâniale de l'aorte dorsale au cours de la 8^e^ semaine d'embryogenèse; l´ACI se déroule au fur et à mesure que l´extrémité dorsale de l´aorte descend dans le thorax. L'échec de ce processus, le développement incomplet ou la croissance excessive de l'ACI peuvent induire une évolution aberrante [4]. Une classification basée sur les données anatomiques et clinico-radiologiques a été rapportée, décrivant l´artère aberrante comme tortueuse, coudée ou enroulée en fonction de sa déviation par rapport au plan vertical [5, 6]. Une aberration de son trajet normal peut placer l'ACI en position latéro et/ou rétropharyngée à proximité immédiate de la paroi pharyngée postérieure [7], c´était le cas de notre patiente dont l´ACI présentait un trajet cervical médialisé et tortueux de façon bilatérale, plus marqué à droite. Des trajets aberrants intracrâniens de l´ACI ont été décrits dans la littérature, notamment dans l´oreille moyenne [8-11]. Le 3^e^ âge est le plus souvent concerné, comme le rapportent Ozturk, Koichi, Yoshinori, Munks et Leong [6, 12-15]. Ces auteurs ont observé des trajets carotidiens internes aberrants chez des patients dont l´âge était compris entre 69 et 82 ans, comme c´était le cas pour notre patiente. L´explication résiderait dans la perte d´élasticité de la paroi vasculaire évoluant graduellement avec l´âge. L'artériosclérose, l'hypertension artérielle et la dysplasie fibromusculaire, qui surviennent aussi avec l'augmentation de l'âge, agissent comme des facteurs induisant une évolution aberrante de l'ACI [5]. Un cas atypique a été rapporté par Allioui *et al*. chez une patiente de 49 ans [2].

De même, le sexe féminin pourrait être plus à risque, du fait de l´action des œstrogènes sur la paroi vasculaire (vasodilatation). Beom Cho-Jun identifie ainsi le sexe féminin comme facteur de risque de distance réduite entre l´ACI et le mur pharyngé, associé à l´âge avancé [16]. Cet auteur recommande un examen pharyngé méticuleux avant toute chirurgie du pharynx chez les patientes âgées. La gêne pharyngée ou dysphagie a été rapportée par plusieurs auteurs comme symptôme révélant une ACI aberrante [2, 3, 6, 17], bien que cette pathologie soit souvent asymptomatique [2, 17]. Le cas que nous rapportons était symptomatique, la patiente se plaignant de toux en quintes évoluant sur le mode chronique. Kathryn *et al*. ont rapporté un cas d´ACI aberrante révélé par la toux et la dysphonie [17]. La toux s´expliquerait ici par le contact de l´artère carotide interne aberrante dans l´hypopharynx avec l´épiglotte; elle serait donc réflexe, pour protéger les voies respiratoires basses. Nous avons observé ce phénomène au cours de la nasofibroscopie chez notre patiente.

Les trajets aberrants de l´artère carotide interne sont bien mis en évidence sur la TDM injectée ou mieux, sur l'IRM réalisée avec des séquences angiographiques [1, 7, 18, 19]; cette dernière est considérée comme l'examen le moins invasif permettant de confirmer l'anomalie et les rapports de l'ACI avec les autres structures du pharynx [1]. Pour notre patiente, nous avons eu recours initialement à une TDM injectée, fortement évocatrice d´une masse vasculaire pharyngée; ce résultat a conduit à la réalisation d´un angioscanner cervical en complément, nous permettant de conclure à un trajet aberrant de l´artère carotide interne.

Des mesures posturales ont été proposées à notre patiente, avec un suivi trimestriel en consultation ORL. L´abstention thérapeutique est rapportée dans la littérature par de nombreux auteurs [2, 15, 17] du fait du risque hémorragique majeur. Une hémiparésie, une aphasie, une surdité, un syndrome de Horner et des vertiges intraitables peuvent également survenir si l´ACI est accidentellement blessée [9]. Le traitement doit être discuté en concertation pluridisciplinaire (chirurgien cervical, chirurgien vasculaire, anesthésiste réanimateur) et le patient associé à la décision thérapeutique après avoir été informé de toutes les modalités et complications possibles. Knox *et al*. rapportent un cas d´ACI aberrante de l´oreille moyenne traité avec succès par sacrifice endovasculaire après des épisodes récurrents d'hémorragie consécutifs à la pose d´aérateurs transtympaniques [10].

## Conclusion

La toux chronique peut révéler un trajet aberrant de l´artère carotide interne, bien que cette entité demeure un diagnostic d´exclusion après un bilan classique bien conduit et non contributif. La présence d´une masse pharyngée chez un patient âgé doit inciter le praticien ORL à la prudence. L'évaluation de tels patients impose la réalisation d´un examen minutieux de la tête et du cou, complété par une imagerie appropriée avant toute biopsie et/ou chirurgie.
